# The Hsp70 inhibitor 2-phenylethynesulfonamide inhibits replication and carcinogenicity of Epstein–Barr virus by inhibiting the molecular chaperone function of Hsp70

**DOI:** 10.1038/s41419-018-0779-3

**Published:** 2018-06-29

**Authors:** Huan Wang, Lang Bu, Chao Wang, Yaqian Zhang, Heng Zhou, Xi Zhang, Wei Guo, Cong Long, Deyin Guo, Xiaoping Sun

**Affiliations:** 1 Department of Pathogen Biology, School of Basic Medical Sciences, Wuhan University, Wuhan, 430071 China; 2 School of Medicine (Shenzhen), Sun Yat-sen University, Guangzhou, 510080 China; 3 Second Clinical College of Wuhan University, Wuhan, 430071 China; 4 Department of Pathology and Physiology, School of Basic Medical Sciences, Wuhan University, Wuhan, 430071 China; 5 The State Key Laboratory of Virology, Hubei Province Key Laboratory of Allergy and Immune-related Diseases, Department of Pathogen Biology, School of Basic Medical Sciences, Wuhan University, Wuhan, 430071 China

## Abstract

Epstein–Barr virus (EBV) can infect cells in latent and lytic period and cause serious disease. Epstein–Barr virus nuclear antigen 1 (EBNA1) is essential for the maintenance of the EBV DNA episome, replication and transcription. 2-phenylethynesulfonamide (PES) is a small molecular inhibitor of Heat shock protein 70 (Hsp70), which can interact with Hsp70 and disrupts its association with co-chaperones and substrate proteins of Hsp70. In our study, we found that PES could decrease the expression of EBNA1, which is independent of effects on EBNA1 transcription or proteasomal degradation pathway. The central glycine–alanine repeats domain was not required for inhibition of EBNA1 expression by PES. Also, PES could reduce the amount of intracellular EBV genomic DNA. PES inhibited proliferation and migration but induced cell cycle arrest and apoptosis of EBV positive cells. In addition, silencing of Hsp70 decreased expression of EBNA1 and the amounts of intracellular EBV genomic DNA, and PES increased this effect on a dose-dependent manner. On the contrast, over-expression of Hsp70 enhanced the expression of EBNA1 and the amounts of intracellular EBV genomic DNA, but PES inhibited this effect on a dose-dependent manner. Furthermore, Hsp70 interacted with EBNA1 but PES interfered this interaction. Our results indicate that PES suppresses replication and carcinogenicity of Epstein–Barr virus via inhibiting the molecular chaperone function of Hsp70.

## Introduction

Epstein–Barr virus (EBV), a human γ-herpesvirus, is an obligate human pathogen that can infect cells in viral latent period and lytic period. In the vast majority of adult population worldwide, EBV can cause a persistent latent infection for life, but at most cases it is well controlled^1,2^. Nevertheless, EBV infection can result in serious disease such as infectious mononucleosis (IM), nasopharyngeal carcinoma (NPC), certain gastric carcinomas, lymphoproliferative disease, Burkitt and Hodgkin lymphoma^3–7^. EBV can cause three types of latent infection, termed as type I, II, and III latency. While Type I or Type III latency are observed in Burkitt’s lymphoma cells, type II latency is found in nasopharyngeal or Hodgkin’s disease cells^8–10^.

Epstein–Barr virus nuclear antigen 1 (EBNA1) is the only protein of EBV-encoded proteins in all forms of EBV-infected cells^2,11^. EBNA1 is essential for the maintenance of the EBV DNA episome, replication, transcription, and postmitotic EBV genome segregation, which are important processes for viral persistence and related oncogenic potential^12,13^. The C-terminal domain of EBNA1 binds to the viral origin of plasmid replication (*Ori*P), which is required for episome maintenance and DNA replication^13,14^. The N-terminal domain of EBNA1 tethers EBV episomes to host cellular mitotic chromosomes and interphase chromatins, which is necessary for the persistence of the episome in propagating cells^14–16^. The central glycine–alanine repeat (GAr) domain of EBNA1 can suppress the translation of its own mRNA and play a key role in the mechanism of immune evasion ^17,18^.

Heat shock proteins (Hsps) are highly-preserved proteins. The Hsps efficiently stable unfolded or misfolded peptides, repair denatured proteins and prevent the accumulation of improperly folded or denatured proteins, thereby protecting normal cells against various harmful stimuli^19–21^. Hsp70 is an ATP-dependent chaperone involving in co-translational and posttranslational protein folding^22^. Hsp70 maintains intracellular homeostasis via binding improperly folded polypeptides, refolds these clients by performing the cycles of co-chaperone accelerated ATP hydrolysis, and then transfers them to Hsp90. In this way, Hsp70 promotes protein transports or posttranslational modifications, and targets improperly folded substrates for degradation ^23,24^.

2-phenylethynesulfonamide (PES), originally identified as a molecule that inhibits p53 binding to mitochondrion, was then shown to be a selective Hsp70 function inhibitor^25^. Specifically, PES acts on the carboxyterminal substrate-binding domain of Hsp70 and disrupts its association with co-chaperones and substrate proteins of Hsp70^26,27^. PES-mediated cell death of tumor cells is associated with the accumulation of misfolded proteins, the impairment of autophagic processes, and the inhibition of lysosomal functions ^26,28,29^.

In this present study, our results indicate that PES inhibits proliferation and migration and causes cell cycle arrest of EBV-positive cells. PES induces apoptosis by inhibiting autophagy in HONE1/Akata and HK1/Akata cells. However, PES increases the expression of LMP1, which may induce apoptosis in B95-8 cells by activating caspase through NF-κB pathway. In addition, PES decreases EBNA1 expression and reduces lytic replication of EBV. Meanwhile, our findings illustrate that PES decreases viral protein and viral genomic DNA via inhibiting the function of Hsp70. Furthermore, our results show that Hsp70 interacts with EBNA1 but PES inhibits this interaction. Finally, PES significantly inhibits the growth of xenografted tumor induced by HONE1/Akata cells in BALB/c nude mice.

## Results

### PES decreases EBNA1 expression independent of effects on EBNA1 transcription or proteasomal degradation

**Fig. 1:**
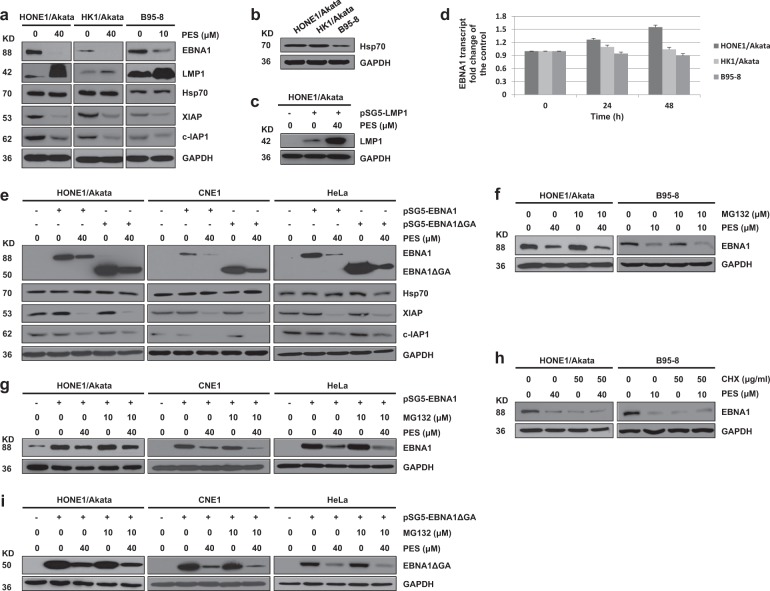
PES decreases EBNA1 expression and the GAr domain is not required for inhibition of EBNA1 expression by PES. **a** HONE1/Akata, HK1/Akata, and B95-8 cells were treated with concentrations of PES as mentioned in Figure for 48 h. **b** HONE1/Akata, HK1/Akata, and B95-8 cells were untreated and examined the natural expression levels of Hsp70. **c** HONE1/Akata cells were transfected with empty vector pSG5or pSG5-LMP1, followed by a 44 h treatment with 40 µM PES beginning at 4 h after transfection. Whole-cell proteins were extracted and subjected to western blotting. **d** HONE1/Akata, HK1/Akata, and B95-8 cells were treated with drug vehicle control or PES (40, 40 or 10 μM) for 24 and 48 h, respectively. Total RNAs were extracted and reversely transcribed to cDNA. The level of EBNA1 transcript was examined by RT-PCR. The level of EBNA1 transcript in cells treated with vehicle control is set as 1. **e** HONE1/Akata, CNE1, and HeLa cells were transfected with empty vector, pSG5-EBNA1, or pSG5-EBNA1ΔGA for 4 h, followed by a 44 h treatment of 40 µM PES. **f** HONE1/Akata and B95-8 cells were treated with indicated concentrations of PES for 48 h in the absence or presence of proteasome inhibitor MG-132 (10 µM) for the last 16 h. **g** HONE1/Akata, CNE1, and HeLa cells were transfected with pSG5-EBNA1 for 4 h, followed by a 44 h treatment with 40 µM PES in the absence or presence of MG-132 (10 µM) at the last 16 h. **h** HONE1/Akata and B95-8 cells were treated with concentrations of PES as mentioned in the figure for 48 h in the absence or presence of CHX (50 μg/ml) at the last 12 h. **i** HONE1/Akata, CNE1, and HeLa cells were transfected with pSG5-EBNA1ΔGA, followed by a 48 h treatment with 40 µM PES beginning at 4 h after transfection in the absence or presence of MG-132 (10 µM) at the last 16 h. Whole-cell extracts were analyzed using western blotting

To determine whether PES alters the expression level of EBNA1, a variety of types of latently infected EBV-positive cells were treated with vehicle drug control or PES. PES decreased EBNA1 expression in every EBV-infected cell line examined (Fig. 1a). As expected, expressions of XIAP and c-IAP1 (obligate Hsp70 clients, which can serve as biomarkers to monitor Hsp70 inhibition^30,31^) were also decreased, whereas Hsp70 expression was not reduced. This confirmed that PES can inhibit the function of Hsp70. In addition, the expression level of LMP1, which is another EBV latent protein, was not decreased, suggesting that the inhibitory effect of PES on EBNA1 was specific (Fig. 1a). As shown in Fig. 1b, the Hsp70 natural expression levels in HONE1/Akata and HK1/Akata cells were higher than the level in B95-8 cells, which may lead to the high sensitivity to EBNA1 inhibition by lower dose of PES in B95-8 cells. To further determine if PES decreases EBNA1 expression, HONE1/Akata cells were transfected with pSG5-LMP1. HONE1/Akata, CNE1 and HeLa cells were transfected with pSG5-EBNA1. These cells were then treated with PES. As shown in Fig. 1c and e, PES significantly decreased the expression of transfected pSG5-EBNA1, whereas expression of pSG5-LMP1 was increased. PES reduced the expression level of EBNA1, however, the mRNA levels of EBNA1 were not significantly decreased after PES treatment, suggesting that PES does not inhibit EBNA1 transcription in HONE1/Akata, HK1/Akata and B95-8 cells (Fig. 1d).

To further examine whether PES decreased the expression of EBNA1 via the proteasome pathway, HONE1/Akata, CNE1 and HeLa cells were transfected with the pSG5-EBNA1 and treated with PES in the absence or presence of the proteosomal inhibitor MG-132. The results showed that PES decreased the expression of endogenous and exogenous EBNA1 to a similar degree in the absence or presence of MG-132 (Fig. 1f, g). In addition, HONE1/Akata and B95-8 cells were treated with PES in the absence or presence of cycloheximide (CHX) (50 μg/ml). As shown in Fig. 1h, CHX did not attenuate the effect of PES on endogenous EBNA1 protein level. These results suggested that PES decreases EBNA1 expression independent of effects on EBNA1 transcription or proteasomal degradation.

### The glycine–alanine repeats (GAr) domain is not required for inhibition of EBNA1 expression by PES

EBNA1 contains a central GAr domain that inhibits the translation of EBNA1 mRNA and play a key role in the mechanism of immune evasion. To determine if the GAr domain is required for the inhibition effect of PES on EBNA1 expression, we compared the effect of PES on the full-length EBNA1 protein or the mutant EBNA1 lacking of the GAr domain (EBNA1ΔGA) protein. HONE1/Akata, CNE1, and HeLa cells were transfected with the pSG5-EBNA1 or pSG5-EBNA1ΔGA, followed by treatment with PES. These results showed that PES had similar inhibition effects on the expression of transfected pSG5-EBNA1 and pSG5-EBNA1ΔGA (Fig. 1e) and MG-132 did not attenuate effect of PES on expression of EBNA1ΔGA protein (Fig. 1i). These results suggested that the GAr domain is not required for the effect of PES on EBNA1.

### PES reduces replication of EBV in HONE1/Akata and B95-8 cells

**Fig. 2:**
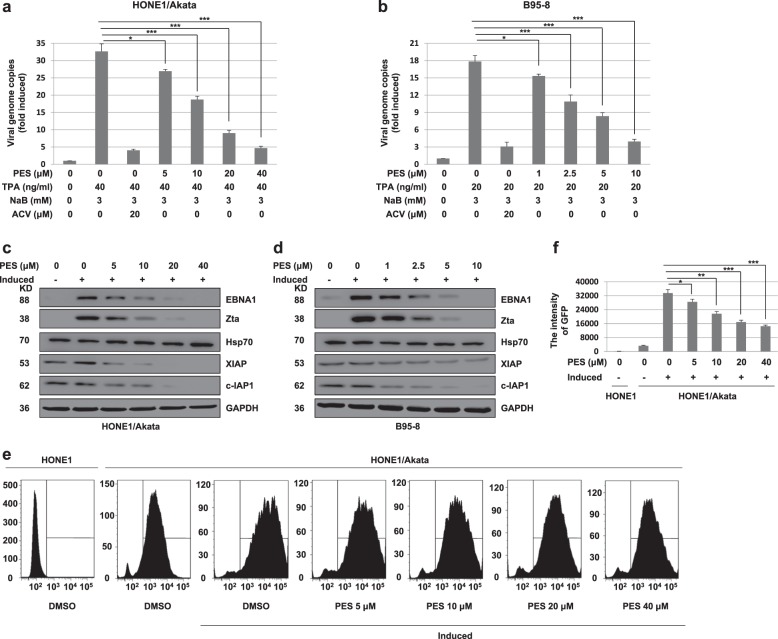
PES reduces lytic replication of EBV in HONE1/Akata and B95-8 cells. **a** HONE1/Akata and **b** B95-8 cells were induced or not by TPA (40 or 20 ng/ml) and NaB (3 mM) for 4 h, followed with a 44 h treatment of ACV or gradient concentrations of PES. The intracellular genomic DNA were prepared and quantified by RT-PCR with EBNA1 primers. Each sample was normalized to the amount of the GAPDH gene. **c** HONE1/Akata and **d** B95-8 cells were induced by TPA and NaB for 4 h, and then treated with indicated concentrations of PES for 44 h. Whole-cell proteins were extracted and analyzed by western blotting. **e**,**f** HONE1/Akata cells were induced by TPA and NaB, followed by a 44 h treatment with increasing concentrations of PES beginning at 4 h after EBV induction. The untreated HONE1 cells and above-treated HONE1/Akata cells were collected and assessed by flow cytometer to analyze the intensity of GFP (*P < 0.05, **P < 0.01, ***P < 0.001)

To examine if PES affects EBV replication, HONE1/Akata and B95-8 cells were treated by TPA (40 or 20 ng/ml) and NaB (3 mM) to induce the lytic viral replication, followed by treatment with PES. The intracellular viral genomic DNA was extracted and the amounts of intracellular viral DNA were determined by RT-PCR. Acyclovir (ACV), a drug effectively reducing production of EBV, was used as a positive drug control^32^. The results shown that ACV and PES significantly reduced the amounts of intracellular viral genomic DNA in induced HONE1/Akata and B95-8 cells (Fig. 2a, b). EBV transcription factors Zta, encoded by the viral immediate early (IE) gene BZLF1, is a lytic switch protein that is necessary for EBV reactivation and lytic DNA replication^33–35^. As shown in Fig. 2c, d, PES reduced expression of EBNA1, Zta, XIAP and c-IAP1 in induced HONE1/Akata and B95-8 cells by a dose-dependent manner, but PES did not affect the expression of Hsp70. Meanwhile, The EBV Akata genome DNA in HONE1/Akata cells was tagged with GFP gene, and these cells could be visualized by flow cytometer analysis. The increase in the intensity of GFP fluorescence corresponds to the increasing intracellular viral genome copy numbers. HONE1/Akata cells treated with induction and PES and the untreated HONE1 cells were collected and assessed by flow cytometer to analyze the intensity of GFP fluorescence. The intensity of GFP fluorescence in HONE1/Akata cells was dose-dependently reduced after PES treatment (Fig. 2e, f). These results indicated that PES reduces EBV intracellular DNA replication in HONE1/Akata and B95-8 cells.

### PES inhibits proliferation and migration of EBV-positive cells

**Fig. 3:**
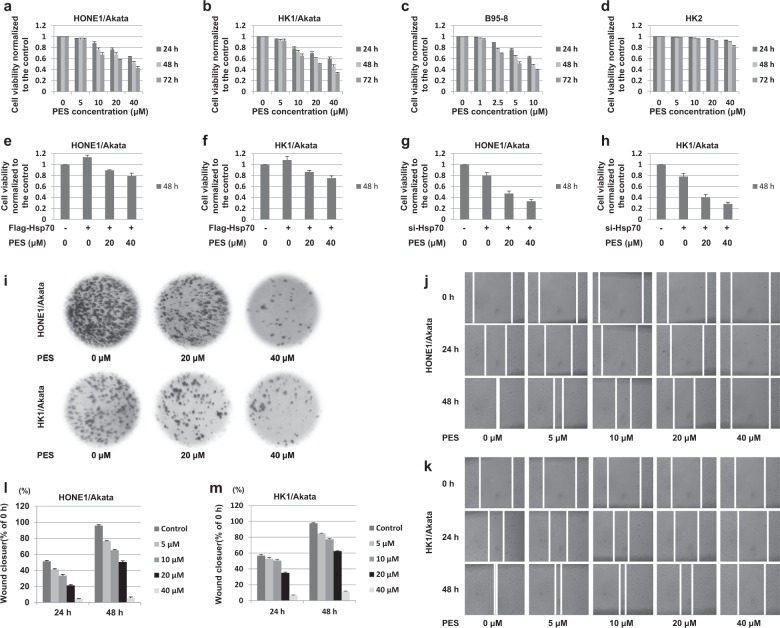
PES inhibits proliferation and migration of EBV-positive cells. **a** HONE1/Akata, **b** HK1/Akata, **c** B95-8 and **d** HK2 cells were treated or not with increasing concentrations of PES for 24, 48, and 72 h, respectively. **e** HONE1/Akata and **f** HK1/Akata cells were transfected with the pEF-Flag-Hsp70, followed by treatment with PES (20, 40 μM) beginning at 4 h after transfection. **g** HONE1/Akata and **h** HK1/Akata cells were transfected with Hsp70 siRNA, followed by treatment with PES (20, 40 μM) beginning at 4 h after transfection. Cell viability was determined by CCK-8 assays. **i** HONE1/Akata and HK1/Akata cells were treated or not with PES (20 or 40 μM) and cultured for another 15 days. Colony formation assay was carried out to determine the long-term effect of PES on cell proliferation. HONE1/Akata (**j**,**l**) and HK1/Akata (**k**,**m**) cells were treated with the linear scratch wounds, followed by the treatment of PES (0, 5, 10, 20 or 40 μM) for 24 and 48 h. Then the migration of the cells towards the wound was visualized. Photographs were captured and analyzed using Image J software

To determine whether PES affects cell viability of EBV-positive cells, HONE1/Akata, HK1/Akata, and B95-8 cells, together with EBV-negative HK2 cells were treated with vehicle drug control (0.006% DMSO) or a series of increasing concentrations of PES for 24, 48, and 72 h. As shown in Fig. 3a–d, PES inhibited the cell viability of HONE1/Akata, HK1/Akata, and B95-8 cells in a dose- and time-dependent manner, but showed a slight proliferation inhibition in HK2 cells. In addition, over-expression of Hsp70 reduced the inhibitory effect of PES on cell proliferation, but silencing of Hsp70 increased the inhibitory effect of PES on cell proliferation in HONE1/Akata and HK1/Akata cells (Fig. 3e–h). To determine the long-term effect of PES on cell proliferation, HONE1/Akata and HK1/Akata cells were treated with vehicle control or PES and cultured for another 15 days. As shown in Fig. 3i, 40 µM PES reduced the levels of colony formation to 10–20% of the levels in HONE1/Akata and HK1/Akata cells treated with vehicle drug control. The results suggested that PES inhibits proliferation of EBV-positive cells efficiently. In order to research further on whether PES affects the migration capability of EBV-positive cells, HONE1/Akata and HK1/Akata cells were treated with the linear scratch wounds, followed by the treatment of vehicle control or the increasing concentrations of PES for 24 and 48 h, respectively. As shown in Fig. 3j–m, PES indeed affects the migration of EBV-positive cells.

### PES induces cell cycle arrest and apoptosis in EBV-positive cells

**Fig. 4:**
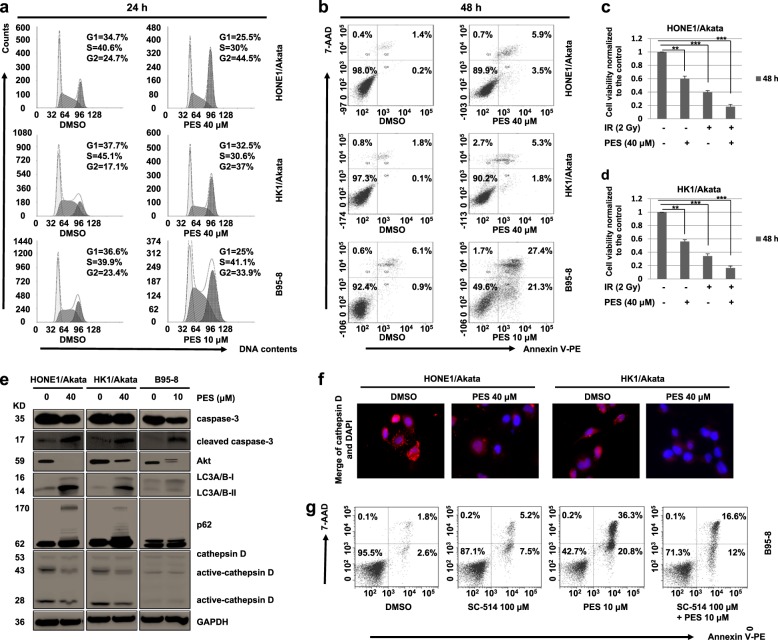
PES induces cell cycle arrest and apoptosis in EBV-positive cells. **a** HONE1/Akata, HK1/Akata, and B95-8 cells were treated with indicated concentrations of PES for 24 h, followed by staining with PI. Cell cycle was assessed by flow cytometer. **b** HONE1/Akata, HK1/Akata and B95-8 cells were treated with concentrations of PES as mentioned above for 48 h and then stained with V-PE/7-AAD. Apoptotic cells analysis was performed using flow cytometer. **c** HONE1/Akata and **d** HK1/Akata cells were treated with 40 μM PES and then irradiated by a single dose of radiation (2 Gy). After 48 h, cell viability was determined by CCK-8 assays. **e** HONE1/Akata, HK1/Akata, and B95-8 cells were treated with concentrations of PES as mentioned above for 48 h. Whole-cell proteins were extracted and subjected to western blotting. **f** HONE1/Akata and HK1/Akata cells were treated with 40 μM PES for 48 h. Then cells were stained with cathepsin D antibody and visualized using the Leica confocal LCS-SP8-STED Nanoscope. **g** B95-8 cells were treated with SC-514 (100 μM) and PES (10 μM) for 48 h and then stained with V-PE/7-AAD. Apoptotic cells analysis was performed using flow cytometer (**P < 0.01, ***P < 0.001)

To determine if PES affects cell cycle, HONE1/Akata, HK1/Akata, and B95-8 cells were treated with vehicle control or PES for 24 h. The results showed that PES arrests cell cycles in G2 phase effectively (Fig. 4a). The ionizing radiation (IR) is one of the most common therapeutic agents to treat EBV-associated malignancies^36^. Meanwhile, it is reported that Hsp70 inhibitors PES could induce G2 arrest in cancer cells^37^. In our study, G2 arrest induced by PES can increase the sensitivity of EBV cells to radiotherapy (Fig. 4c, d). Then we examined if PES induces EBV-positive cell apoptosis. As shown in Fig. 4b, PES resulted in significant cell apoptosis in HONE1/Akata, HK1/Akata, and B95-8 cells. The western blotting results also showed that PES can increase the expressions of cleaved caspase-3 and decrease the expression of Akt, which indicates that PES can inhibit the proliferation and promote the apoptosis of EBV positive cells (Fig. 4e).

Autophagy includes microautophagy, macroautophagy, and chaperone-mediated autophagy (CMA). Hsp70, as an important regulator of apoptotic signaling pathways, plays a key role during CMA^38,39^. To determine if PES induces apoptosis by inhibiting autophagy, we examined expression of autophagy markers p62, LC3A/B, and the lysosomal cysteine peptidase cathepsin D. PES caused accumulation of p62 and LC3A/B-II and significantly reduced expression of active-cathepsin D in HONE1/Akata and HK1/Akata cells (Fig. 4e). Immunofluorescence assays results showed that cathepsin D is located in the lysosomes and staining of cathepsin D indicated the presence of punctate structures in HONE1/Akata and HK1/Akata cells without PES treatment. After PES treatment, the staining became diffuse, indicating that cathepsin D is relocated into the cytosol, which is consistent with the results of Western blotting (Fig. 4f). These results showed that PES induces apoptosis by inhibiting autophagy in HONE1/Akata and HK1/Akata cells. However, there were no significant changes in the expressions of LC3A/B, p62, and cathepsin D in B95-8 cells. It is reported that expression of LMP1 in B95-8 cells could induce programmed cell death by a way depending on activation of NF-κB pathway, whereas LMP1 in EBV-positive NPC cells, including HONE1/Akata and HK1/Akata cells, does not induce cell death^40^. Our results showed that PES treatment causes increased expressions of LMP1 in HONE1/Akata, HK1/Akata and B95-8 cells (Fig. 1a). The increased expression of LMP1 may be the most important factor leading to apoptosis of B95-8 cells. It was shown that SC-514, which inhibits NF-κB activation, attenuates the apoptosis induced by PES in B95-8 cells (Fig. 4g). The results showed that PES increases expression of LMP1, which may induce apoptosis in B95-8 cells by activating caspase through NF-κB pathway.

### Hsp70 increases the expression level of EBNA1, but PES inhibits this effect on a dose-dependent manner

**Fig. 5:**
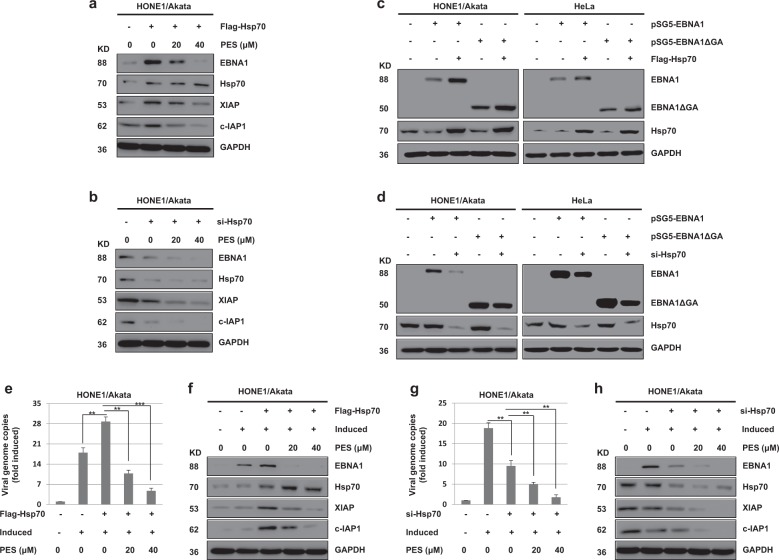
**The change of EBNA1 expression induced by Hsp70 is not associated with the GAr domain, and Hsp70 promote the lytic replication of EBV in HONE1/Akata cells but PES inhibits this effect.** HONE1/Akata cells were transfected with pEF-Flag-Hsp70 (**a**) or Hsp70 siRNA (**b**) for 24 h, followed by treatment with PES (20 or 40 μM) for 48 h. **c** HONE1/Akata and HeLa cells were transfected with pEF-Flag-Hsp70 for 24 h, followed by transfection with pSG5-EBNA1 or pSG5-EBNA1ΔGA into above cells for 48 h. **d** HONE1/Akata and HeLa cells were transfected with Hsp70 siRNA for 24 h, followed by transfection with pSG5-EBNA1 or pSG5-EBNA1ΔGA into above cells for 48 h. Whole-cell proteins above were extracted and subjected to western blotting. HONE1/Akata cells were induced by TPA and NaB for 4 h, and then transfected with pEF-Flag-Hsp70 (**e**) or Hsp70 siRNA (**g**), followed by treatment with PES beginning at 4 h after transfection. The intracellular genomic DNA were quantified by RT-PCR with EBNA1 primers. Each sample was normalized to the amount of the GAPDH gene. **f**,**h** Whole-cell proteins above were extracted and analyzed using western blotting (**P < 0.01, ***P < 0.001)

To investigate the effect of Hsp70 on EBNA1 expression, HONE1/Akata cells were transfected with pEF-Flag-Hsp70 or Hsp70 siRNA, followed by treatment with PES (20 or 40 μM). The results showed that over-expression of Hsp70 increases the expressions of endogenous EBNA1, XIAP, and c-IAP1, but PES inhibits this effect on a dose-dependent manner (Fig. 5a). Meanwhile, knock-down of Hsp70 decreased the expressions of endogenous EBNA1, XIAP, and c-IAP1, but PES increased this effect (Fig. 5b). In addition, we determined if the GAr domain is required for the change of EBNA1 expression up-regulated by Hsp70. The results suggested that the changes of EBNA1 expression induced by Hsp70 are not associated with the GAr domain in HONE1/Akata and HeLa cells (Fig. 5c, d).

### Hsp70 promotes the lytic replication of EBV, but PES inhibits this effect on a dose-dependent manner

To further determine whether Hsp70 affects the lytic replication of EBV, HONE1/Akata cells were induced by TPA and NaB, and then transfected with pEF-Flag-Hsp70 or Hsp70 siRNA, followed by treatment with PES. The copy numbers of the intracellular viral DNA and the expression of EBNA1 were increased in the induced HONE1/Akata cells transfected with Hsp70, but PES inhibited this effect on a dose-dependent manner (Fig. 5e, f). In addition, the copy numbers of the intracellular viral DNA and the expression of EBNA1 in the induced HONE1/Akata cells transfected with Hsp70 siRNA were decreased, but PES increased this effect on a dose-dependent manner (Fig. 5g, h). These results indicated that Hsp70 promotes replication of EBV, but PES inhibits this effect on a dose-dependent manner in HONE1/Akata cells.

### EBNA1 interacts with Hsp70, but PES interferes this interaction

**Fig. 6:**
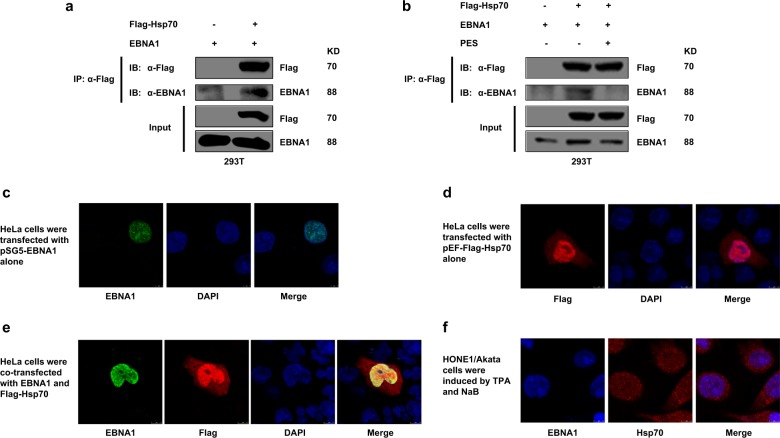
EBNA1 interacts with Hsp70 but PES interferes this interaction. **a** 293T cells were transfected with pEF-Flag-Hsp70 in the presence of pSG5-EBNA1. At 48 h post-transfection, IP assays were performed with Flag antibody, and then immunoprecipitated with the indicated antibodies. **b** 293T cells were transfected with pSG5-EBNA1 and pEF-Flag-Hsp70, separately or together, followed by treatment with the vehicle drug control or PES (20 μM) beginning at 4 h after transfection. 48 h later, IP assays were performed. HeLa cells were transfected with pSG5-EBNA1 (**c**) and pEF-Flag-Hsp70 alone (**d**) and co-transfected with pSG5-EBNA1 and pEF-Flag-Hsp70 (**e**). **f** HONE1/Akata cells were induced by TPA and NaB. At 48 h post-transfection or post-induction, cells were fixed, stained with antibodies, and then visualized using the Leica confocal LCS-SP8-STED Nanoscope

The results of co-immunprecipitation assays showed that EBNA1 interacts with Hsp70 but PES interferes this interaction (Fig. 6a, b). To further confirm the cellular localization of EBNA1 and Hsp70, immunofluorescence assays were performed. The results showed that a strong signal from EBNA1 overlapped with the Hsp70 signal in HeLa cells (Fig. 6c–e). Meanwhile, co-location of EBNA1 and Hsp70 was also found in induced HONE1/Akata cells (Fig. 6f).

### In vivo effects of PES on BALB/c nude mice inoculated with HONE1/Akata cells

**Fig. 7:**
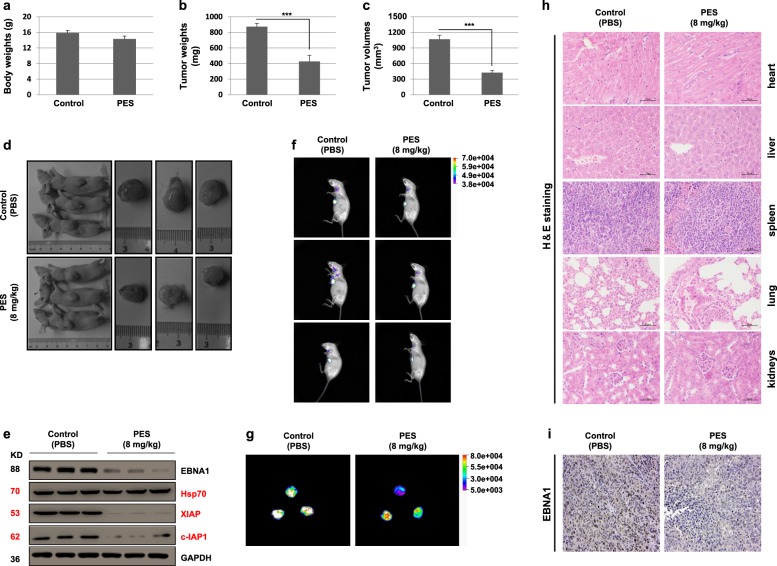
PES inhibits the growth of tumor in vivo. BALB/c nude mice with hypodermic tumors were treated with vehicle control (PBS) or 8 mg/kg PES for 5 days. Body weights (**a**), tumor weights (**b**), and tumor volumes (**c**) were evaluated in above BALB/c nude mice. The whole values are shown as mean ± SD of six mice. **d** Representative images of mice treated with PBS or PES and tumors in above mice are shown. **e** Lysates of tumor tissues were analyzed by western blotting. **f**, **g** The intensity of GFP in tumors of above mice were assessed by In-vivo Xtreme Imaging System. **h** The five important organs (heart, liver, spleen, lung, and kidneys) of above mice were detected by H&E staining. **i** The tumor tissues were detected by immunohistochemical staining using antibody against EBNA1. Randomly selected micrographs were given (***P < 0.001)

Since the above-mentioned results showed the effects of PES on cells in vitro, the possible effects of PES in vivo were also investigated. To study if PES influences the growth of the EBV-positive NPC in BALB/c nude mice, 1 × 10^7^ HONE1/Akata cells were subcutaneously inoculated into the armpit of the mice. After seven days, the mice were injected intraperitoneally with a dose of 8 mg/kg PES or control PBS per day for consecutive five days and then euthanized. The dose of PES in vivo in this study has been reported to be safe^41,42^. PES treatment had no significant effect on the body weights of mice (Fig. 7a). The tumor weights and volumes of the PES-treated group were significantly smaller than the PBS-treated group (Fig. 7b, c). Representative images of mice and tumors in above mice are shown in Fig. 7d. Western blotting results of the tumor tissues indicated that PES significantly down-regulates the expression of EBNA1, XIAP, and c-IAP1, whereas Hsp70 expression is not reduced (Fig. 7e). In addition, the intensity of GFP fluorescence in tumors with PBS treatment were stronger than that in tumors with PES treatment (Fig. 7f, g). The results of H&E staining showed that PES has no significant negative effect on mice (Fig. 7h). Immunohistochemical staining showed that the number of EBNA1-positive cells in the tumor tissues treated with PES were decreased (Fig. 7i). These results suggested that, in vivo, PES significantly inhibits the growth of tumors induced by EBV-positive HONE1/Akata cells.

## Discussion

It has been reported that EBV infection is significantly associated with increased risk and poor prognosis of EBV-related diseases. EBNA1 is essential for EBV episome maintenance, transcription and replication, which are mediated by EBNA1 binding to homologous *OriP* DNA^12^. This study has shown that the Hsp70 inhibitor PES effectively inhibited proliferation of EBV-positive cells in vitro and in vivo. The PES-induced G2 arrest could increase sensitivity of EBV cells to radiotherapy. PES induces apoptosis by inhibiting autophagy in HONE1/Akata and HK1/Akata cells. However, PES increases the expression of LMP1, which may induce apoptosis in B95-8 cells by activating caspase through NF-κB pathway. Meanwhile, PES inhibited the expression of EBNA1 in various cell lines. But these effects were not related with EBNA1 transcription and proteasomal degradation. Furthermore, the GAr domain was also not necessary for inhibition of EBNA1 expression by PES. Our findings suggested that PES down-regulates expression of EBNA1 by a mechanism related with Hsp70.

Our study has shown that PES reduced replication of EBV in HONE1/Akata and B95-8 cells. The inhibition of Hsp70 significantly reduced the viral protein synthesis and virus replication, and PES increased this effect on a dose-dependent manner. On the contrary, the overexpression of Hsp70 induced the viral protein synthesis and virus replication, but PES inhibited this effect on a dose-dependent manner. The results of EBV infected cells treated with Hsp70 inhibitor PES in this study demonstrated that Hsp70 activity was required for efficient production of EBV, which is consistent with a previous study^43^, suggesting that Hsp70 could be considered as a positive cellular factor for EBV infection.

There is growing evidence that Hsp70 plays essential roles in replication of many viruses, such as α-herpesvirus HSV-1^44^, β-herpesvirus HCMV^45^ and γ -herpesvirus KSHV^46^, suggesting that Hsp70 may be playing important roles in EBV lytic infection. EBV encodes eight proteins in latent stage, including the nuclear proteins EBNA-1, -2, -3A, -3B, -3C, -5, and the membrane proteins LMP-1, -2A, and -2B. The EBNA genes belong to the same transcription unit and the different mRNAs are generated by alternative splicing from a large primary transcript. EBNA3A was reported to not only interact with the chaperones Hsp70 and the co-chaperones Hsp40 but also up-regulate their expression levels^47^. The nucleolus localization of Hsp70 was enhanced with the presence of EBNA-5^48^. It has been reported that LANA1 in KSHV can interact with Hsp70^49^. Although the full sequences of LANA1 and EBNA1 do not have similarities, they have similar structures and functions. Therefore, we speculated that there may also be some correlation between Hsp70 and EBNA1. Through various experiments, our study determined that Hsp70 interacts with EBNA1 to regulate EBV protein synthesis and viral replication, and that PES inhibits this interaction. It is reported that Hsp70 translocates from cytoplasm to nucleoplasm in response to stressful treatment, such as heavy metals, heat shock, ultraviolet radiation and viral infections^50,51^. In our experiments, most of transfected Hsp70 co-located with EBNA1 in nucleus of HeLa cells. In HONE1/Akata cells induced by TPA and NaB, endogenous Hsp70 also co-located with EBNA1 in the nuclear, which may be related to the stimuli of EBV. The interaction between Hsp70 and EBNA1 may explain the inhibitory effect of PES on EBV replication.

As drug resistance is a major obstacle to antiretroviral therapy, the effectiveness of antiviral drugs is thus severely limited^52^. Fortunately, drug-resistance did not emerge when Hsps inhibitors were used to block numerous viruses replication, suggesting that the Hsps inhibitors might become attractive candidates for cure of viruses-caused human disease^53^. Previous studies have shown that Hsp70 knockout in mice did not cause pathological changes in animals, suggesting that reducing Hsp70 levels in cells appears to be relatively safe^54,55^. In our study, after treatment of PES, the organs of the BALB/c nude mice showed no abnormal lesions. Meanwhile, PES significantly inhibited the proliferation of HONE1/Akata cell-induced xenograft tumors in BALB/c nude mice and decreased the expression of EBNA1 in these tumors. Notably, these results highlight the therapeutic potential of PES for the treatment of EBV-related diseases.

Altogether, our study reveals the potential of the novel Hsp70 inhibitor PES to inhibit EBV replication and growth of EBV-associated tumors. Therefore, Hsp70 inhibitor PES may provide a novel therapeutic approach for the treatment of EBV-associated malignancies ^46^.

## Materials and methods

### Cell lines, reagents, and antibodies

EBV-positive cells HONE1/Akata and HK1/Akata were generous gifts given by Prof. S.W. Tsao at the University of Hong Kong in China. These two cell lines were made by introducing EBV Akata genome DNA that contains GFP gene into NPC cell lines HONE1 and HK1 cells. EBV-positive B lymphoma cell line B95-8 and EBV-negative NPC cell line CNE1 were kind gifts from Prof. Y. Cao (Central South University, Changsha, China). EBV-negative NPC cell line HONE1 was purchased from the American Type Culture Collection (ATCC; VA, USA). EBV-negative cell line HK2, a human renal tubular epithelial cell, was kindly provided by Professor L. Zheng (Wuhan University, Wuhan, China). HeLa and 293T cells were kindly provided by Professor H. Li (Wuhan University, Wuhan, China). HONE1, B95-8, and CNE1 cells were cultured in RPMI 1640 medium (Hyclone, USA) containing 10% fetal bovine serum (FBS; Gibco-BRL, Gaithersburg, MD, USA). HeLa and 293T cells were maintained in DMEM medium (Hyclone, USA) containing 10% FBS. For maintaining the recombinant EBV genomes, HONE1/Akata and HK1/Akata cells were cultured in RPMI 1640 medium containing 10% FBS and 0.4 μg/ml Geneticin (G418). HK2 cells were maintained in DME/F-12 containing 10% FBS and hEGF (Sigma-Aldrich, St. Louis, MO, USA).

PES (Merck Millipore, MA, USA), MG-132 (Merck Millipore, MA, USA), cycloheximide (CHX; Sigma-Aldrich, St. Louis, MO, USA), 12-O-tetradecanoylphorbol-13-acetate (TPA; Sigma-Aldrich, St. Louis, MO, USA), and acyclovir (ACV; Selleck Chemicals, Shanghai, China), SC-514 (Sigma-Aldrich, St. Louis, MO, USA) were dissolved in dimethylsulfoxide (DMSO; Sigma-Aldrich, St. Louis, MO, USA). Sodium butyrate (NaB; Sigma-Aldrich, St. Louis, MO, USA) was dissolved in PBS.

The antibodies used in this study were given as follows: anti-caspase-3 (#9668), anti-cleaved caspase-3 (#9664), anti-Akt (#4685), anti-XIAP (#14334), anti-c-IAP1 (#7065), anti-cathepsin D (#2284), anti-LC3A/B (#4108), anti-p62 (#88588), and anti-GAPDH (#5174) were purchased from Cell Signaling Technology. Anti-EBNA1 (sc-81581) and anti-Zta (sc-53904) were purchased from Santa Cruz. Anti-LMP1 (ab78113), EBV nuclear antigen (ab8329), goat anti-mouse IgG (ab6789), and goat anti-rabbit IgG (ab6721) were purchased from Abcam. Anti-Hsp70 (ADI-SPA-810-D) was purchased from Enzo. Anti-Flag (CFLKT002) was purchased from Beijing Chunfenglv Biomedical Technology (Co., Ltd., Beijing, China). Goat anti-mouse Alexa Fluor 555 (A21422), Goat anti-rabbit Alexa Fluor 555 (A21428) and goat anti-rabbit FITC (F-2765) were purchased from Invitrogen. Goat anti-mouse DyLight 405 (A23110) was purchased from Abbkine.

### Cell viability and colony formation assay

Cell viability assays were performed according to the instructions, which was described previously^56^. As for colony formation assay, cells were digested into cells suspension with trypsin solution. A total of 4 ml of single cell suspension was seeded onto 6 cm plates at a density of 200 cells/ml. After adherence, cells were treated with PES for 48 h and cultured for another 15 days. Then cells were stained with glutaraldehyde crystal violet mixture (Sigma-Aldrich) for 30 min and washed with tap water carefully. After the plates with colonies being dried, digital images were taken.

### Wound-healing assay

Cells were grown to confluence on 6 well plates. The linear scratch wounds were created using a 10 μl tip, and then the cells were incubated with culture medium containing 2.5% FBS. After that, all the cells were treated with PES. Finally, images of the cells along the scrape line were captured using microscope after wounding 0, 24, and 48 h, respectively. The wound-healing capacity was analyzed by measuring the changes in the width of the wound gaps using Image J software.

### Flow cytometer analysis

Cell cycle was measured using the Annexin V/PI apoptosis kit (MultiSciences, China) and cell apoptosis were measured using the Annexin V-Phycoerythrin (PE)/7-amino-actinomycin D (7-AAD) apoptosis detection kit (MultiSciences, China) according to the manufacturer’s directions. Then cells were immediately analyzed by the flow cytometer (BD FACSAria III, BD) in the Research Center for Medicine and Structural Biology, Wuhan University.

### X-ray irradiation

Cells were grown to confluence on 6 well plates. Then the cells were irradiated at a distance of 100 cm at room temperature by X-ray produced using a Varian medical linear accelerator (Zhongnan Hosptial). X-irradiation was carried out at a dose rate of 2 Gy/min.

### Plasmids

The plasmids pSG5 vector, pSG5-EBNA1, and pSG5-EBNA1ΔGA have been described previously^57^. The plasmid pEF-Flag-Hsp70 was constructed by inserting the Hsp70 sequence into the pEF vectors. The plasmids were isolated using the plasmid DNA extraction kit (cat no. CFLKP001-50) purchased from Beijing Chunfenglv Biomedical Technology (Co., Ltd., Beijing, China). The primers were given as follows: pEF-Flag-Hsp70, forward, 5′-CGGCTAGCATGTCGGTGGTGGGCATAGACCTGG-3′ and reverse, 5′-GCTCTAGACTAGTTCTTCAAATAATTTGGTCGT-3′.

### Cell transfection

For transfection of DNA, cells were transfected with plasmids using X-treme GENE HP DNA Transfection reagent (Roche) according to the manufacturer’s protocol. For siRNA transfection, cells were transfected with Hsp70 siRNA and control-siRNA molecules (Ribobio, China) using Lipofectamine RNAiMAX (Invitrogen). The siRNA primers were as follows: Hsp70 siRNA forward, 5′-AGAAGAAGGUGCUGGACAAdTdT-3′ and reverse, 5′-dTdTUCUUCUUCCACGACCUGUU-5′ ^58^.

### Quantitative real-time PCR

Methods for extracting cDNA and genomic DNA have been previously described^56^. Expression level of EBNA1 mRNA and the amounts of intracellular viral genomic DNA were quantified by the CFX96 real-time PCR detection system using a SYBR Premix Ex Taq kit (Takara). The quantitative real-time PCR conditions were: a 30 s-denaturation step at 95 °C, followed by 40 cycles of 10 s at 95 °C, 10 s at 60 °C and 15 s at 72 °C. Melting curve analysis was performed from 65–95 °C (with 0.5 °C increments). All data were normalized to the control gene encoding GAPDH. The primers were given as follows: EBNA1 forward, 5′-TCATCATCATCCGGGTCTCC-3′ and EBNA1 reverse, 5′-CCTACAGGGTGGAAAAATGGC-3′^2^; GAPDH forward, 5′-ACATCGCTCAGACACCATG-3′ and GAPDH reverse, 5′-TGTAGTTGAGGTCAATGAAGGG-3′ ^56^.

### Co-immunoprecipitation assays and western blotting

293T cells (5 × 10^6^) seeded in 10 cm dishes were transfected with plasmids for 48 h. The cells were harvested and lysed by lysis buffer (50 mM Tris-HCl, pH 7.6, 150 mM NaCl, 10 mM NaF, 2 mM EGTA, 1 mM Na3VO4, 0.5% Triton-X-100, and 2 mM DTT) containing cocktail for 30 min on ice. After centrifugation at 12,000 g for 20 min at 4 °C, the supernatants were collected and incubated with the Protein A/G PLUS-Agarose beads and appropriate primary antibody or IgG isotype control antibody at 4 °C overnight. After washing four times, these compounds were mixed with 2× loading buffer and boiled for 5 min at 100 °C. The immune complexes were resolved on 10% SDS–PAGE gels and subjected to western blotting that was performed according to the instructions as indicated ^56^.

### Immunofluorescence assays

HeLa cells were grown in chamber slides and transfected with plasmids. HONE1/Akata cells grown in chamber slides were induced by TPA and NaB. These cells were fixed with 4% paraformaldehyde for 10 min and permeabilized with 0.2% Triton X-100 for 10 min. Then cells were incubated with blocking buffer (5% BSA in PBS) for 30 min at 37 °C, incubated with appropriate primary antibodies diluted in diluent buffer (1% BSA in PBS) overnight at 4 °C and washed four times with PBST (0.3% Tween 20 in PBS). Next, the cells were incubated with appropriate second antibodies diluted in diluent buffer for 1 h at 37 °C. Finally, the nuclear staining was conducted with 4, 6- diamidino-2-phenylindole (DAPI) for 5 min. Confocal images were captured using the Leica confocal LCS-SP8-STED nanoscopes (Leica, Germany).

### Animal studies

The procedures and protocols of animal experiments were approved by the Medical Ethics Committee of Wuhan University. BALB/c nude mice were purchased and maintained in the Animal Experiment Center of Animal Biosafety Level-III Laboratory of Wuhan University. HONE1/Akata cells (1 × 10^7^) were subcutaneously inoculated into the armpit of the 6-week-old male BALB/c nude mice. After seven days, when tumors had grown to be palpable, the mice were injected intraperitoneally with a dose of 8 mg/kg PES or control PBS per day for consecutive five days. Then the mice were euthanized and the body weights, tumor weights and volumes were measured. The GFP intensity of tumors were assessed by the In-vivo Xtreme Imaging System (BrukerXtreme BI, USA). Hematoxylin and eosin (H&E) staining and immunohistochemistry analysis were performed according to the method described in a previous study ^59^.

### Statistical analysis

The data were shown as the mean ± standard deviation (mean ± SD) from at least three independent experiments. The statistical significance of the difference between any two samples was evaluated by Student’s t-test using GraphPad Prism for Windows version 5.0 (GraphPad Software, USA). The values of *P* *<* 0.05 were considered statistically significant.
